# Predictive Factors for a Long Hospital Stay in Patients Undergoing Laparoscopic Cholecystectomy

**DOI:** 10.1155/2017/5497936

**Published:** 2017-01-23

**Authors:** Wasana Ko-iam, Trichak Sandhu, Sahattaya Paiboonworachat, Paisal Pongchairerks, Anon Chotirosniramit, Narain Chotirosniramit, Kamtone Chandacham, Tidarat Jirapongcharoenlap, Sunhawit Junrungsee

**Affiliations:** ^1^Clinical Epidemiology, Faculty of Medicine, Chiang Mai University, Chiang Mai, Thailand; ^2^Department of Surgery, Faculty of Medicine, Chiang Mai University, Chiang Mai, Thailand; ^3^Department of Anesthesiology, Faculty of Medicine, Chiang Mai University, Chiang Mai, Thailand; ^4^Department of Surgery, Bumrungrad International Hospital, Bangkok, Thailand

## Abstract

*Background*. Although the advantages of laparoscopic cholecystectomy (LC) over open cholecystectomy are immediately obvious and appreciated, several patients need a postoperative hospital stay of more than 24 hours. Thus, the predictive factors for this longer stay need to be investigated. The aim of this study was to identify the causes of a long hospital stay after LC.* Methods*. This is a retrospective cohort study with 500 successful elective LC patients being included in the analysis. Short hospital stay was defined as being discharged within 24 hours after the operation, whereas long hospital stay was defined as the need for a stay of more than 24 hours after the operation.* Results*. Using multivariable analysis, ten independent predictive factors were identified for a long hospital stay. These included patients with cirrhosis, patients with a history of previous acute cholecystitis, cholangitis, or pancreatitis, patients on anticoagulation with warfarin, patients with standard-pressure pneumoperitoneum, patients who had been given metoclopramide as an intraoperative antiemetic drug, patients who had been using abdominal drain, patients who had numeric rating scale for pain > 3, patients with an oral analgesia requirement > 2 doses, complications, and private ward admission.* Conclusions*. LC difficulties were important predictive factors for a long hospital stay, as well as medication and operative factors.

## 1. Introduction

Laparoscopic cholecystectomy (LC) is one of the most common minimally invasive elective surgeries. The advantages of LC over open cholecystectomy (OC) have been immediately appreciated. These include an earlier return of bowel function, less postoperative pain, improved cosmesis, a shorter length of hospital stay, an earlier return to full activity, and decreased overall cost [1–4]. Nevertheless, there are several patients who have had a postoperative hospital stay of more than 24 hours, due to conversion to open surgery or complications. Thus, the factors predicting this should be investigated to inform the at-risk patients. The aim of this study was to identify the causes of long hospital stay after LC. From these results, a guideline for patient selection for further ambulatory surgery might be offered in the future.

## 2. Patients and Methods

The present study was approved by the ethics committee of the Faculty of Medicine, Chiang Mai University. In this retrospective cohort study, 500 patients who underwent elective LC at Chiang Mai University Hospital between July 2010 and June 2014 were recruited. All 500 successful LC procedures were included in the analysis. The medical records of the patients were reviewed systematically. They were divided into two groups: long hospital stay (LS group) and short hospital stay (SS group). A short hospital stay was defined as returning home within 24 hours of the operation, whereas a long hospital stay was defined as a stay of more than 24 hours after the operation. Variables were documented from medical records for comparison between the two groups. A total of 34 variables were identified for comparison. These included 20 patient variables: age, gender, American Society of Anesthesiologists (ASA) risk classification, comorbidities (including diabetes mellitus, hypertension, dyslipidemia, cirrhosis, cardiovascular disease, chronic kidney disease, thalassemia, and other factors such as asthma, COPD, and thyroid diseases), body mass index, previous upper intra-abdominal surgery, indications of surgery, and patients on anticoagulation with warfarin; eight operative variables: surgeons' status, type of procedure, use of preemptive analgesia, intraoperative antiemetic drugs, intraoperative cholangiogram, operative time, operative findings of thickened gallbladder wall, presence of adhesions, incidental perforation of gallbladder, and use of abdominal drain; and six postoperative variables: postoperative nausea or vomiting (PONV), numeric rating scale (NRS) pain score, parenteral analgesia requirement, oral analgesia requirement, complications, and type of ward admission.

### 2.1. Anesthetic Technique

Balanced general anesthesia with endotracheal intubation was performed in all patients. Intravenous induction was performed using thiopental (5 mg/kg body weight), and intravenous fentanyl (1 *μ*g/kg body weight) was given as an analgesic.

### 2.2. Surgical Technique

The American technique (surgeon standing on the side) was used in all patients. Then, standard LC was performed by the set of surgeons.

### 2.3. Postoperative Treatment

At the end of the surgery, each patient received one kind of intravenous antiemetic drug including the combination of dexamethasone 8 mg and metoclopramide 10 mg [5], ondansetron 8 mg [6], or metoclopramide 10 mg alone. The score of PONV in each patient was recorded with the number 0 or 1 (0 = no nausea or vomiting, 1 = nausea or vomiting). In case of nausea and vomiting, intravenous metoclopramide 10 mg was given as a rescue drug.

A numeric rating scale (NRS) (0–10) was used to assess the postoperative pain score. Morphine 0.05 mg/kg was administered intravenously by a staff nurse as a rescue analgesic as the patient required (if NRS ≥ 6). After 2 hours postoperatively, if NRS was 3–5, pain was controlled by oral paracetamol with codeine (15 mg) 1-2 tablets and then repeated every 4–6 hours in case of minor pain.

In addition, some patients received preemptive analgesia with etoricoxib 120 mg orally two hours before surgery at the ward.

### 2.4. Statistical Analysis

Statistical comparison of these 34 variables was performed using Fisher's exact test for categorical variables, *t*-test for normally distributed continuous variables, and Mann–Whitney *U* test for non-normally distributed continuous variables. The pre- and postoperative factors identified as significant were included in a multivariable analysis by log risk regression to identify the predictive factors for a long hospital stay. A *p* value of 0.05 was considered as being statistically significant. STATA version 11.0 was used for data analysis.

## 3. Results

Of the 500 laparoscopic cholecystectomies, 411 (82.20%) could be discharged within 24 hours after operation, while 89 (17.80%) had a longer hospital stay. The postoperative stay was 1 day and 2 days (range: 2–19) in the SS group and LS group, respectively (*p* < 0.001). Reasons for long hospital stay included observation of postoperative fever (*n* = 19), surgery related causes (*n* = 42; postoperative pain = 34, delayed oral diet = 1, retained abdominal drain = 4, and postoperative complications = 3), PONV (*n* = 10), medical causes (*n* = 13), and patient preference (*n* = 5). Postoperative complications included bowel injury (*n* = 2) and septicemia (*n* = 1). The patients who stayed due to observation of postoperative fever were discharged uneventfully (Table 1).

The patient factors that were significantly associated with a long hospital stay included ASA risk classification (*p* < 0.001), history of cirrhosis (*p* = 0.039), and being on anticoagulation with warfarin (*p* < 0.001) (Table 2). In addition, several operative and postoperative factors were also associated with a long hospital stay including types of intraoperative antiemetic drug (*p* = 0.021), intraoperative cholangiogram (*p* = 0.037), operative time (*p* = 0.010), incidental perforation of the gallbladder (*p* = 0.005), use of an abdominal drain (*p* < 0.001), PONV (*p* = 0.008), postoperative pain (*p* < 0.001), parenteral analgesia requirement (*p* = 0.001), oral analgesia requirement (*p* < 0.001), and complications (*p* = 0.005) (Tables 3 and 4).

Sixteen potential factors were identified in the comparison between the SS and LS groups in the univariable analysis (Table 5). The factors that increased the risk of a long hospital stay included patients with an ASA class 3, a history of previous acute cholecystitis, cholangitis, or pancreatitis, a history of cirrhosis, being on long-term anticoagulation with warfarin, having standard-pressure pneumoperitoneum (14 mmHg), having been given metoclopramide as an intraoperative antiemetic drug, having an intraoperative cholangiogram, having an operative time of more than 60 minutes, having an incidental perforation of the gallbladder, using an abdominal drain, PONV, an NRS pain score more than 3, a parenteral analgesia requirement of more than 2 doses, an oral analgesia requirement of more than 2 doses, complications, and private ward admission.

The multivariable analysis showed that 10 independent predictive factors indicated a long hospital stay (Table 6): patients with a history of cirrhosis, patients with a history of previous acute cholecystitis, cholangitis, or pancreatitis, patients on long-term anticoagulation with warfarin, patients with standard-pressure pneumoperitoneum (14 mmHg), patients who had been given metoclopramide as an intraoperative antiemetic drug, using an abdominal drain, having an NRS pain score of more than 3, having an oral analgesia requirement of more than 2 doses, complications, and private ward admission.

## 4. Discussion and Conclusions

Improvement in LC and anesthetic techniques, together with increased familiarity with the procedure, has led to progressively shorter hospital stays [7]. However, two studies have reported that LC patients fulfilling the following criteria had a significant association with longer hospital stays: patients aged more than 60 years, patients with ASA class 3, patients with complicated gallstones, patients with increased operative time, patients with intraoperative findings of thickened gallbladder wall, and patients with adhesions and perforations of the gallbladder [8, 9].

Our results showed that the independent predictive factors for a long hospital stay were a history of cirrhosis, a history of previous acute cholecystitis, cholangitis, or pancreatitis, being on long-term anticoagulation with warfarin, having standard-pressure pneumoperitoneum (14 mmHg), having been given metoclopramide as an intraoperative antiemetic drug, using an abdominal drain, having an NRS pain score of more than 3, having an oral analgesia requirement of more than 2 doses, complications, and private ward admission.

It is widely accepted that patients with liver cirrhosis are at higher risk of developing complications to surgical procedures, and the condition will result in a longer hospital stay of between 3 and 6.9 days (average 2.8 days) [10]. There are some technical difficulties with performing LC in patients with cirrhosis [11]. The cirrhotic liver parenchyma is stiff due to fibrous transformation and could interfere with the frequently standard maneuver in LC where retraction of the gallbladder fundus is performed to expose the triangle of Calot [12]. It has therefore been suggested that laparoscopic procedures in patients with liver cirrhosis are performed with a lower intra-abdominal pressure [10].

Previous acute cholecystitis, cholangitis, and pancreatitis all contributed to the longer hospital stay. These conditions are associated with inflammation of the right upper quadrant abdomen and cause the distortion of anatomy such as biliary fibrosis. These make the surgery difficult and result in prolonged operative time.

Despite being minimally surgically invasive, laparoscopic surgery has yet to be proven safe in patients receiving anticoagulants. In the present study, warfarin was discontinued preoperatively in all cases. Heparin anticoagulation was individualized according to each patient's risk of thrombosis [13]. LC was completed in each patient without resulting hemorrhagic complications, but a longer hospital stay was required for the continuation of postoperative anticoagulation treatment.

Postoperative pain is an important practical problem in LC. A postoperative pain score of more than 3 was one of the predictive factors of a longer hospital stay. Oral analgesia consumption, which reflected the degree of postoperative pain, especially an oral analgesia requirement of more than 2 doses, was another predictive factor of a longer hospital stay. 78.65% (70/89) of patients in the long hospital stay group had a longer hospital stay due to a postoperative pain score of more than 3. Satisfactory pain control involved a multimodality of treatment, which included low-pressure pneumoperitoneum (7 mmHg) [14, 15], preemptive analgesia [15, 16], intraoperative local anaesthesia infiltration, and postoperative analgesia.

Several studies have shown that low-pressure pneumoperitoneum is feasible and safe and results in reduced postoperative pain compared with standard-pressure pneumoperitoneum [14, 15, 17, 18]. Our results confirmed the potential benefits of the reduced length of hospital stay when the procedure of low-pressure pneumoperitoneum was used, whereas standard-pressure pneumoperitoneum resulted in a longer hospital stay.

Although PONV was not an independent risk factor for delayed hospital stays, it was a significant risk factor in the univariable analysis in our study. Since PONV was not actively assessed after the operation, its incidence depended a great deal on complaints by the patients. Thus, inadequate documentation with underestimation of the incidence of PONV would be expected in this retrospective analysis. PONV is known to be a frequent and distressing source of discomfort during the postoperative period, especially after laparoscopic procedures, with an incidence rate as high as 70%. The use of proper antiemetic drugs during the operation might also reduce the incidence of PONV [5, 19, 20].

Based on the evidence, ondansetron is more effective than metoclopramide in preventing PONV after LC [6, 21]. Furthermore, the administration of dexamethasone combined with metoclopramide had significant effects in the prophylaxis of nausea and vomiting after LC and also shortened the hospital stay [5]. Our results from the present study confirmed that the potential effect of the combination of dexamethasone with metoclopramide is better than of ondansetron and metoclopramide for preventing PONV and shortening the hospital stay after LC.

The use of abdominal drain significantly increased the hospital stay after LC. At least 48 hours were needed before removing the drain. In addition, the complications in the present study that occur during surgery (bowel injury and bile duct injury) or after surgery (septicemia) also take several days for treatment and postoperative administration of antibiotics.

In our setting, there are two types of wards for patients undergoing LC, that is, general and private wards. Private wards were a popular type because there are privacy and comfort. For this reason, the patients in private wards were satisfied and wanted to stay in the postoperative period for more than one day.

However, our results also suggested that while ASA class 3, intraoperative cholangiogram, operative time of more than 60 minutes, incidental perforation of the gallbladder, parenteral analgesia requirement of more than 2 doses, and PONV were significant risk factors in the univariable analysis, they were not independent risk factors for long hospital stays in multivariable analysis. Nevertheless, these patients should be of concern as regards complications and also a potential extended hospital stay.

To avoid unnecessary longer hospital stays, intraoperative cholangiogram should be performed in only selected cases such as patients at risk for bile duct injury or patients with anomaly of bile duct. Long hospital stay was also correlated with prolonged operative time and complications in difficult cases. Thus, surgeons should be cautious about the complications in these conditions, especially the injury of the bile duct.

Although it has been found that LC in elderly patients is associated with higher complication rates and longer hospital stays [22], these findings were contradicted in our study. Our results showed that age was not associated with a longer hospital stay. In addition, our results also showed that gender, BMI [23, 24], previous upper intra-abdominal surgery history, surgeons' status, thickened gallbladder wall, and the presence of adhesions were not associated with delayed hospital stay.

In evaluating patient-related risk factors, comorbidities such as diabetes mellitus, hypertension, dyslipidemia, cardiovascular disease, chronic kidney disease, and thalassemia were also analyzed. Patients with diabetes mellitus are known to have poorer surgical outcomes and higher rates of intraoperative complications [25]. On the other hand, our results showed that more patients with diabetes mellitus and other comorbidities belonged to the short stay group. Nevertheless, careful sugar control during the perioperative course should be considered for patients with diabetes mellitus.

The limitations of this study included the bias inherent to the retrospective nature of the design. Inadequate documentation, such as underestimation of the incidence of PONV, resulted in PONV being insignificant in multivariable analysis. However, a huge patient cohort and carefully adjusted variables in the analysis method provide interesting results of influencing factors. In addition, most of our results still were concurrent with previous findings from other studies.

Our study was a retrospective review of elective LC during a 4-year period at one center. The study included all patients who underwent successful elective LC surgery without open conversion. With broad inclusion criteria, a more accurate assessment of any influencing factors for a longer hospital stay was expected. With proper patient selection and adequate preoperative preparation, more patients could benefit from a reduced postoperative hospital stay.

In terms of patient factors, patients with cirrhosis, on long-term anticoagulation medication with warfarin, or with a history of acute cholecystitis, cholangitis, or pancreatitis should be informed of the possibility of a lengthened postoperative hospital stay. In addition, the use of low-pressure pneumoperitoneum (7 mmHg) and a combination of dexamethasone and metoclopramide as intraoperative antiemetic drugs during elective LC may be helpful in reducing the length of postoperative hospital stay.

From these results, patients with ASA class 1 or 2, patients without cirrhosis, and patients with uncomplicated gallstones could be enrolled for further ambulatory LC. The proper procedure and management of PONV and postoperative pain might help to generate further ambulatory LC in our country.

## Figures and Tables

**Table 1:** Reasons for long hospital stay in long stay group.

Reasons for long hospital stay	LS group (*N* = 89)
Observation of postoperative fever	19
Surgery related causes	
Postoperative pain	34
Delayed oral diet	1
Retained abdominal drain	4
Postoperative complications	
Bowel injury	2
Septicemia	1
Postoperative nausea or vomiting	10
Medical causes	13
Patient preference	5

**Table 2:** Patients' variables.

Patients' variables, *n* (%)	LS group (*n* = 89)	SS group (*n* = 411)	*p* value
Age (years), mean ± SD	55.44 ± 11.75	53.55 ± 13.10	
≤60 years	61 (16.85)	301 (83.15)	0.363
>60 years	28 (20.29)	110 (79.71)	
Gender			
Male	25 (15.06)	141 (84.94)	0.321
Female	64 (19.16)	270 (80.84)	
ASA risk classification			
ASA class 1	29 (18.13)	131 (81.88)	<0.001
ASA class 2	47 (15.02)	266 (84.98)	
ASA class 3	13 (48.15)	14 (51.85)	
Comorbidities			
Diabetes mellitus	12 (18.18)	54 (81.82)	1.000
Hypertension	35 (19.13)	148 (80.87)	0.547
Dyslipidemia	16 (16.84)	79 (83.16)	0.882
Cirrhosis	8 (36.36)	14 (63.64)	0.039
Cardiovascular disease	8 (30.77)	18 (69.23)	0.108
Chronic kidney disease	5 (29.41)	12 (70.59)	0.202
Thalassemia	2 (8.70)	21 (91.30)	0.400
Others (asthma, COPD, thyroid diseases, etc.)	33 (18.86)	142 (81.14)	0.713
BMI (kg/m^2^), mean ± SD	24.21 ± 4.16	24.63 ± 12.39	
Normal weight (18.5–24.9)	51 (16.56)	257 (83.44)	0.639
Overweight (25–29.9)	30 (19.11)	127 (80.89)	
Obese (30–39.9)	8 (24.24)	25 (75.76)	
Morbidly obese (≥40)	0	2 (100.00)	
Previous upper intra-abdominal surgery			
Yes	11 (19.30)	46 (80.70)	0.716
No	78 (17.61)	365 (82.39)	
Indication for surgery			
Symptomatic gallstones	84 (18.06)	381 (81.94)	0.818
Gallstones with previous ERCP	21 (21.88)	75 (78.13)	0.239
Gallstones with previous cholangitis or pancreatitis	12 (22.64)	41 (77.36)	0.343
Gallstones with previous acute cholecystitis	21 (25.93)	60 (74.07)	0.055
Gallbladder polyp	4 (15.38)	22 (84.62)	1.000
Acute cholecystitis	0	1 (100.00)	1.000
On long-term anticoagulation with warfarin			
Yes	7 (77.78)	2 (22.22)	<0.001
No	82 (16.70)	409 (83.30)	

SS: short stay; LS: long stay; ASA: American Society of Anesthesiologists; BMI: body mass index; ERCP: endoscopic retrograde cholangiopancreatography.

**Table 3:** Operative variables.

Operative variables, *n* (%)	LS group (*n* = 89)	SS group (*n* = 411)	*p* value
Surgeons' status			
Surgical attending	36 (16.22)	186 (83.78)	0.480
Resident	53 (19.06)	225 (80.94)	
Type of procedure			
Low-pressure pneumoperitoneum (7 mmHg)	39 (15.42)	214 (84.58)	0.163
Standard-pressure pneumoperitoneum (14 mmHg)	50 (20.42)	197 (79.76)	
Use of preemptive analgesia			
Yes	11 (11.96)	81 (88.84)	0.131
No	78 (19.12)	330 (80.88)	
Intraoperative antiemetic drug			
Combination of dexamethasone and metoclopramide	3 (6.00)	47 (94.00)	0.021
Ondansetron	73 (18.25)	327 (81.75)	
Metoclopramide	13 (26.00)	37 (74.00)	
Intraoperative cholangiogram			
Yes	4 (50.00)	4 (50.00)	0.037
No	85 (17.28)	407 (82.72)	
Operative time (min), mean ± SD	79.25 ± 33.11	65.24 ± 22.12	
≤60 min	33 (13.31)	215 (86.69)	0.010
>60 min	56 (22.22)	196 (77.78)	
Operative findings			
Thickened gallbladder wall	16 (25.00)	48 (75.00)	0.116
Presence of adhesions	11 (25.58)	32 (74.42)	0.208
Incidental perforation of gallbladder	25 (28.74)	62 (71.26)	0.005
Use of abdominal drain			
Yes	5 (100.00)	0	<0.001
No	84 (16.97)	411 (83.03)	

SILC: single incision laparoscopic cholecystectomy.

**Table 4:** Postoperative variables.

Postoperative variables, *n* (%)	LS group (*n* = 89)	SS group (*n* = 411)	*p* value
Postoperative nausea or vomiting			
Yes	35 (25.55)	102 (74.45)	0.008
No	54 (14.88)	309 (85.12)	
NRS pain score, mean ± SD	4.62 ± 1.30	3.29 ± 1.22	
≤3	19 (6.74)	263 (93.26)	<0.001
>3	70 (32.11)	148 (67.89)	
Parenteral analgesia requirement (dose), median (range)	1 (0–5)	1 (0–4)	
≤2 doses	56 (14.66)	326 (85.34)	0.001
>2 doses	33 (27.97)	85 (72.03)	
Oral analgesia requirement (dose), median (range)	3 (0–10)	1 (0–3)	
≤2 doses	22 (6.73)	305 (93.27)	<0.001
>2 doses	67 (38.73)	106 (61.27)	
Complications			
Yes	3 (100.00)	0	0.005
No	86 (17.30)	411 (82.70)	
Type of ward admission			
Private ward	70 (19.94)	281 (80.06)	0.056
General ward	19 (12.75)	130 (87.25)	

NRS: numeric rating scale.

**Table 5:** Potential factors from the univariable analysis.

Factors	Risk ratio	95% confidence interval	*p* value
Preoperative factors			
ASA class 3	3.00	1.92–4.67	<0.001
History of cirrhosis	2.15	1.19–3.86	0.011
History of previous acute cholecystitis, cholangitis, or pancreatitis	1.73	1.18–2.53	0.005
On long-term anticoagulation with warfarin	4.66	3.12–6.96	<0.001
Perioperative factors			
Type of procedure			
Low-pressure pneumoperitoneum (7 mmHg)	1.00		
Standard-pressure pneumoperitoneum (14 mmHg)	1.31	0.90–1.92	0.161
Intraoperative antiemetic drug			
Combination of dexamethasone and metoclopramide	1.00		
Ondansetron	3.04	1.00–9.30	0.051
Metoclopramide	4.33	1.31–14.30	0.016
Intraoperative cholangiogram	2.89	1.41–5.95	0.004
Operative time > 60 min	1.67	1.13–2.47	0.011
Incidental perforation of gallbladder	1.85	1.24–2.77	0.003
Use of abdominal drain	5.89	4.85–7.16	<0.001
Postoperative factors			
Postoperative nausea or vomiting	1.72	1.18–2.51	0.005
NRS pain score > 3	4.77	2.96–7.67	<0.001
Parenteral analgesia requirement > 2 doses	1.91	1.31–2.78	0.001
Oral analgesia requirement > 2 doses	5.76	3.99–8.99	<0.001
Complications	5.78	4.77–7.01	<0.001
Private ward admission	1.56	0.98–2.50	0.062

**Table 6:** Predictive factors from multivariable analysis.

Predictive factors	Adjusted risk ratio	95% confidence interval	*p* value
Preoperative factors			
History of cirrhosis	2.29	1.63–3.22	<0.001
History of previous acute cholecystitis, cholangitis, or pancreatitis	1.53	1.08–2.15	0.015
On long-term anticoagulation with warfarin	1.94	1.10–3.40	0.021
Perioperative factors			
Type of procedure			
Low-pressure pneumoperitoneum (7 mmHg)	1.00		
Standard-pressure pneumoperitoneum (14 mmHg)	1.48	1.03–2.14	0.034
Intraoperative antiemetic drug			
Combination of dexamethasone and metoclopramide	1.00		
Ondansetron	2.80	0.97–8.07	0.056
Metoclopramide	3.60	1.17–11.11	0.026
Postoperative factors			
NRS pain score > 3	2.38	1.48–3.82	<0.001
Oral analgesia requirement > 2 doses	3.77	2.44–5.80	<0.001
Use of abdominal drain	4.04	2.16–7.56	<0.001
Complications	3.50	1.12–10.98	0.032
Private ward admission	1.75	1.16–2.65	0.008
